# Supplementary material for: Preliminary steps of the development of a Minimum Uniform Dataset applicable to the international wheelchair sector
Source: PLoS One. 2020 Sep 11;15(9):e0238851. doi: 10.1371/journal.pone.0238851 (PMC7485892; doi:10.1371/journal.pone.0238851)
Supplement: S1 File — (DOCX) [file pone.0238851.s001.docx]

S1 File. ISWP Member Survey Questions

Question 1: demographics information

- Name
- Company/organization
- City/town
- State/Province
- Country
- Email

Question 2: Please describe the scope of your organization (i.e. an organization may only provide repairs, maintenance and technical support to clinical services and do no clinical services themselves and/or vice versa)

Question 3: **What occupation best describes you**

Answer options:

- Clinician – hospital based
- Clinician – community-based
- Researcher
- NGO
- Manufacturer
- Other

Question 4: Do you currently collect data on the wheelchair skills abilities of your users?

Answer options: Yes / No

Question 5: What data do you currently collect through your work? Please select all that apply.

| No. of wheelchairs/products delivered |
| --- |
| Type of wheelchairs/products delivered |
| Location of wheelchair users |
| Gender of wheelchair users |
| Age of wheelchair users |
| Disability/Impairment of wheelchair users |
| User feedback or satisfaction |
| Number or type of product repairs |
| Impact on mobility |
| Number or type of product faults |
| Impact on social inclusion |
| Number of follow up consultations |
| Quality of service provision |
| Income or economic situation of wheelchair users |
| Impact on health |
| Impact on income or employment |
| Other (please specify) |

Question 6: How do you collect and/or store this information? Please select all that apply. Please also note in the comments box if there are any specific data collection tools/satisfaction surveys/outcome measurement you use, or if you are using any new technology, software or devices.

Answer options:

| User records - paper |
| --- |
| User interviews - at time of delivery or follow up |
| User interviews - home visits |
| Partner distribution or service reports |
| Spreadsheets to store records |
| Shipping or production records |
| User records - electronic medical/health records |
| Phone conversations |
| Database software |
| User satisfaction, feedback or impact surveys |
| Focus groups or workshops |
| Online database |
| Online forms to submit or record data |
| Mobile application |
| Social media |
| SMS messaging services |
| Other (please specify) |

Question 7: Would you be willing to share de-identified data with an ISWP working group which will help to develop common data fields?

Answer options: Yes/No/Maybe

Question 8: If you answered "NO" or "MAYBE" in the previous question, what are your main concerns about sharing data with the working group.

Question 9: In the future, would you be interested in collecting and sharing data using a standardized data management system, such as a shared online database?

Answer options: Yes/No/Maybe

Question 10: Please indicate the level of importance of each of the following features for a potential standard electronic data collection and sharing system.

Answer options: Very Important/Important/Neutral/Unimportant/Very unimportant

Features:

| Integration with service management procedures (i.e. storing service data for use by staff in day to day user management, stock control, etc) |
| --- |
| Easy to use interface (i.e. little training required) |
| Reports, to allow you to easily create reports, charts or graphs from the data set about your organization's impact |
| Technology designed for developing countries |
| Scalability, to allow you to collect as few or as many data types as you would like |
| Confidentiality of data (i.e. removing the name of your organization when data is shared) |
| Benchmarking, to allow you to compare your data with that of other organizations |

Question 11: If data were collected from a large number of organizations and shared across organizations in an aggregated fashion — with no specific information revealed — would your organization use this data to

Answer options:

| Plan for future work or organizational direction |
| --- |
| Collaborate effectively with other organizations |
| Evaluate the impact of our work |
| Support funding applications |
| Evaluate or quantify our contribution to the sector |
| Benchmark our performance with other organizations |
| Other (please specify) |

Question 12: After reviewing the Wheelchair Service Training Programme Basic and Intermediate forms as well as your own personal development of uniform/minimum datasets (U/MDS), would you conclude that the following should be included as part of a U/MDS:

Answer options:

| Date of Assessment |
| --- |
| Posture support devices |
| Date of Outcome Measurement |
| Reason for referral |
| Date of Fitting |
| Date of Follow-up Outcome Measurement |
| Gender |
| Race |
| User Year of Birth |
| Country |
| Primary Diagnosis |
| Zip Code |
| User Employment |
| User Living Situation |
| Funding Source |
| Environment |
| Hours per day using wheelchair |
| Type of Mobility Device |
| Age of current equipment |
| Make and Model Number, if applicable |
| Current Device Accessories (such as seat elevator, tilt-in-space, recline, elevating legrests, standing) |
| Type of Cushion |
| Make and Model Number, if applicable |
| Time for Assessment (minutes) |
| Time for fitting (minutes) |
| Other (please specify) |

Question 13: Who is involved in the fitting of the device? (select all that apply)

Answer options: Clinician/Technician/Manufacturer Representative
