# Supplementary material for: Preliminary steps of the development of a Minimum Uniform Dataset applicable to the international wheelchair sector
Source: PLoS One. 2020 Sep 11;15(9):e0238851. doi: 10.1371/journal.pone.0238851 (PMC7485892; doi:10.1371/journal.pone.0238851)
Supplement: S2 File — (DOCX) [file pone.0238851.s002.docx]

S2 File. ISWP Minimum Uniform Dataset Bahasa Indonesia Translation.

Baseline Interview

Demographic Information

Informasi Demografi

1. What is your birth date? Close-ended ISWP

Berapa usia Anda?

1. What is your gender? Close-ended ISWP

- Male
- Female
- Other:

Apa jenis kelamin Anda?

- Pria
- Perempuan
- Lainnya:

1. What is your religion? Close-ended

- Hindu
- Moslem
- Catholic
- Christian
- Buddha
- Confucius
- Other :

Apa agama Anda?

- Hindu
- Islam
- Katolik
- Kristen
- Budha
- Konghucu
- Lainnya:

1. What is the highest level of education you completed? Close-ended ISWP

- Some Primary School
- Primary School
- Some Secondary School
- Secondary School
- Some High School
- High School
- Vocational high school
- Some college/university
- College/University
- Advanced Degree
- None
- Do not know

Apa pendidikan terakhir Anda?

- Tidak tamat SD
- SD
- Tidak tamat SMP
- SMP
- Tidak Tamat SMA
- SMA
- SMK
- Tidak tamat Perguruan Tinggi
- Perguruan Tinggi
- Pasca sarjana
- Tidak Sekolah
- Tidak tahu

Disability And Equipment

Disabilitas Dan Peralatan

### Why do you need a mobility aid or aids (help moving or walking)? Wheelchair? Close-ended ISWP

### Hint: (Check all that apply)

- Amputation
- Osteogenesis imperfecta
- Spinal Cord Injury
- Brain Injury
- Polio
- Spinal Tuberculosis
- Cerebral Palsy
- Spina Bifida
- Stroke
- Muscular Dystrophy
- Other:

Mengapa Anda membutuhkan alat bantu gerak atau alat bantu (untuk bergerak atau berjalan)? Kursi roda? (Jawaban bisa lebih dari satu)

- Amputasi (Amputation)
- Tulang rapuh karena cacat bawaan
- Cedera tulang belakang
- Cedera kepala
- Polio
- Tuberkulosis pada tulang belakang
- Kelumpuhan pada otak
- Spina Bifida
- Stroke
- Otot yang lemah karena cacat bawaan
- Lainnya:

1. What year did you receive this diagnosis? Close-ended ISWP

Kapan anda didiagnosis penyakit tersebut?

1. What mobility aids do you currently have? Close-ended (Please check all that apply.) ISWP

- Manual wheelchair
- Powered wheelchair
- Cycle
- Walking stick or cane
- Crutch or crutches
- Walker or walking frame
- Braces
- Artificial leg/legs
- Board with wheels or other homemade device
- No mobility aids

Alat bantu gerak apa yang saat ini Anda gunakan?

- Kursi roda manual
- Kursi roda otomatis
- Sepeda
- Tongkat untuk berjalan
- Tongkat ketiak
- *Walker* atau *walking frame*
- Braces
- Kaki palsu
- Papan dengan roda atau alat buatan sendiri lainnya
- Tidak ada alat bantu mobilitas

1. What mobility aid or aids do you currently use indoors and outside? (Please check all that apply.) Close-ended ISWP

Alat bantu gerak atau alat bantu apa yang Anda gunakan saat ini, baik di luar rumah maupun di dalam rumah? (berikan tanda pada alat-alat yang digunakan)

| **Indoors**  **Dalam rumah** | **Outside**  **Di luar rumah** | **Both Indoors and Outside**  **Di dalam dan di luar rumah** |  |
| --- | --- | --- | --- |
|  |  |  |  |
|  |  |  | Manual wheelchair: Could be active use, sport/recreational, manual postural support, lever propelled, manual assistant-­‐controlled or power-­‐assisted manual wheelchair  **Kursi roda manual:** untuk pengguna yang aktif, olah raga/hiburan, penyangga tubuh, lever propelled, dengan bantuan baik secara manual maupun tidak |
|  |  |  | Powered wheelchair: Could be power wheelchair with postural support or scooter  **Kursi roda otomatis :** tenaganya menggunakan anggota badan atau scooter |
|  |  |  | Cycle: Bicycle, tricycle or quadricycle  **Sepeda:** roda dua, roda tiga roda empat |
|  |  |  | Walking stick or cane  Tongkat untuk berjalan |
|  |  |  | Crutch or crutches  Tongkat ketiak |
|  |  |  | Walker or walking frame |
|  |  |  | Braces |
|  |  |  | Artificial leg or legs  Kaki palsu |
|  |  |  | Board with wheels or other homemade device  Papan dengan roda atau alat buatan sendiri lainnya |
|  |  |  | Other (describe below)  Lainnya (Jelaskan) |

1. About how long have you been using each of your current mobility aids? Close-ended ISWP

(Hint: Ask client to provide a number, if possible, not a range. Include number in one of the columns below).

Sudah berapa lama Anda menggunakan tiap-tiap alat bantu gerak tersebut? (Tanyakan berapa lama, jika memungkinkan bukan perkiraan. Isikan jawabannya dalam kolom di bawah ini)

|  | **How Long Used**  **Lama pemakaian** | | | |
| --- | --- | --- | --- | --- |
|  | **insert number below**  **isikan angkanya di bawah ini** | | | |
| **Current Mobility Aid**  **Alat bantu yang digunakan saat ini** | **Less than 1 year**  **Kurang dari 1 tahun** | **1‐3**  **Years**  **1-3 tahun** | **4‐5**  **Years**  **4-5 tahun** | **More than 5 Years**  **Lebih dari 5 tahun** |
| Manual wheelchair  Kursi roda manual |  |  |  |  |
| Power wheelchair  Kursi roda otomatis |  |  |  |  |
| Cane/crutch/walker  Tongkat ketiak |  |  |  |  |
| Braces |  |  |  |  |
| Artificial leg/legs  Kaki palsu |  |  |  |  |
| Board with wheels or other homemade device  Papan dengan roda atau alat buatan sendiri lainnya |  |  |  |  |
| Other (describe below)  Lainnya (Jelaskan) |  |  |  |  |

1. About how many hours per day and days per week do you use each of your mobility aids? (Interviewer Instructions: Ask client to provide a specific number, if possible. For example, if person says 10 hours, note the number 10 in the column under 9 to 12 hours.) Close-endedISWP

Berapa jam dalam sehari dan berapa hari dalam seminggu Anda menggunakan alat bantu tersebut? (Tanyakan jumlahnya secara lebih spesifik, jika memungkinkan. Contoh, jika 10 jam masukkan dalam kolom 9-12 jam)

|  | **a. Number of Days per Week**  **Jumlah hari per minggu** | | | |  | **b. Number of Hours per Day**  **Jumlah jam per hari** | | | | |
| --- | --- | --- | --- | --- | --- | --- | --- | --- | --- | --- |
| **Current Mobility Aid** | **<1** | **1-3** | **4-6** | **Everyday** |  | **<1** | **1-3** | **4-6** | **7-8** | **9+** |
| Manual wheelchair  Kursi roda manual |  |  |  |  |  |  |  |  |  |  |
| Power wheelchair  Kursi roda otomatis |  |  |  |  |  |  |  |  |  |  |
| Cane/crutch/walker  Tongkat ketiak |  |  |  |  |  |  |  |  |  |  |
| Artificial leg/legs  Kaki palsu |  |  |  |  |  |  |  |  |  |  |
| Board with wheels or other homemade device  Papan dengan roda atau alat buatan sendiri lainnya |  |  |  |  |  |  |  |  |  |  |
| Other (describe below)  Lainnya (Jelaskan) |  |  |  |  |  |  |  |  |  |  |
| No mobility aids used currently  Tidak menggunakan alat bantu gerak saat ini |  |  |  |  |  |  |  |  |  |  |

1. Where did you get each of the assistive devices? Open-ended ISWP

Dari mana Anda mendapatkan semua alat bantu tersebut?

1. Have you ever had a wheelchair before? Close-ended

- Yes
- No

Apakah Anda pernah memiliki kursi roda sebelumnya?

- Ya
- Tidak

1. How long ago did you receive a wheelchair? Open-ended

Berapa lama Anda menerima kursi roda tersebut?

1. What type of wheelchair did you have? Open-ended

Apa jenis kursi roda yang Anda miliki?

1. Where did you get that wheelchair?Open-ended

Dari mana Anda mendapatkan kursi roda tersebut?

1. How come you no longer have a wheelchair?Open-ended

Kenapa Anda tidak memiliki kursi roda lagi?

1. How long have you been without a wheelchair?Open-ended

Berapa lama Anda tanpa kursi roda?

1. Besides this wheelchair, how many other wheelchairs have you had? Close-ended

- 0
- 1
- 2
- 3
- 4
- 5+

Selain kursi roda yang Anda miliki ini, Berapa banyak kursi roda yang pernah Anda miliki?

- 0
- 1
- 2
- 3
- 4
- 5+

Usage

Penggunaan

1. Where do you currently use your wheelchair? (Check all that apply) Close-ended ISWP

### Home

- School
- Sports

### Leisure activities

- Work
- Other public places outside home

### Transportation

- Outdoors on rough surfaces
- Do not know/No Answer

Kemana sajakah biasanya Anda menggunakan kursi roda? (Tandailah setiap jawaban di bawah ini)

- Rumah
- Sekolah
- Tempat olah raga
- Tempat hiburan
- Tempat bekerja
- Tempat umum di luar rumah
- Di sarana transportasi
- Di luar rumah pada jalan yang kasar
- Tidak tahu/Tidak Menjawab

1. About how far do you travel each day in your wheelchair? Close-ended ISWP

- < 100 m
- 100-499m
- 500 m – 999 m
- 1-5 km
- > 5 km
- Do not know

Berapa jauh Anda bepergian setiap harinya dengan menggunakan kursi roda?

- < 100 m
- 100-499m
- 500 m – 999 m
- 1-5 km
- > 5 km
- Tidak tahu

1. Does the wheelchair meet your needs? Close-ended ISWP

- Yes
- No
- Do not know

Apakah kursi roda Anda sesuai dengan kebutuhan Anda?

- Ya
- Tidak
- Tidak tahu

1. Is the wheelchair in good working order and safe to use? Close-ended ISWP

- Yes
- No
- Do not Know

Apakah kursi roda Anda bekerja dengan baik dan aman untuk digunakan?

- Ya
- Tidak
- Tidak tahu

1. Does the wheelchair fit correctly? Close-ended ISWP

- Yes
- No
- Do not Know

Apakah kursi roda anda sesuai dengan ukuran anda?

- Ya
- Tidak
- Tidak tahu

1. Does the wheelchair provide proper fit and postural support? Close-ended ISWP

- Yes
- No
- Do not Know

Apakah kursi roda anda dapat dipakai dengan nyaman?

- Ya
- Tidak
- Tidak tahu

1. Is the cushion in good working order and safe to use? Close-ended ISWP

- Yes
- No
- Do not Know

Apakah alas duduk pada kursi roda anda nyaman dan aman digunakan?

- Ya
- Tidak
- Tidak tahu

1. Does your wheelchair meet your environmental conditions? Close-ended ISWP

- Yes
- No
- Do not Know

Apakah kursi roda Anda sesuai dengan kondisi lingkungan Anda?

- Ya
- Tidak
- Tidak tahu

1. How would you rate your satisfaction with your wheelchair from 1-5? (1 is not satisfied and 5 is very satisfied) Close-ended ISWP

Berapa tingkat kepuasan yang Anda berikan pada kursi roda Anda saat ini? (1 adalah tidak memuaskan dan 5 adalah sangat memuaskan)

1. Where do you anticipate using your new wheelchair? Close-ended

### Home

- - School
  - Sports

### Leisure activities

- - Work
  - Other public places outside home

### Transportation

- - Outdoors on rough surfaces
  - Do not know/No Answer

Kemana sajakah Anda akan menggunakan kursi roda yang baru? (Tandailah setiap jawaban di bawah ini)

o Rumah

o Sekolah

o Tempat olah raga

o Tempat hiburan

o Tempat bekerja

o Tempat umum di luar rumah

o Di sarana transportasi

o Di luar rumah pada jalan yang kasar

o Tidak tahu/Tidak Menjawab

1. How often do you anticipate using your new wheelchair? Close-ended ISWP

Berapa jam dalam sehari dan berapa hari dalam seminggu kira-kira Anda akan menggunakan kursi roda yang baru?

|  | 1. **Number of Hours per Day**   **Jumlah jam per hari** | | | | |  | 1. **Number of Days per Week**   **Jumlah hari per minggu** | | | |
| --- | --- | --- | --- | --- | --- | --- | --- | --- | --- | --- |
|  | **<1** | **1-3** | **4-6** | **7-8** | **>8** |  | **<1** | **1-3** | **4-6** | **Every day**  **Setiap hari** |
| New wheelchair  Kursi roda yang baru |  |  |  |  |  |  |  |  |  |  |
