# Supplementary material for: Preliminary steps of the development of a Minimum Uniform Dataset applicable to the international wheelchair sector
Source: PLoS One. 2020 Sep 11;15(9):e0238851. doi: 10.1371/journal.pone.0238851 (PMC7485892; doi:10.1371/journal.pone.0238851)
Supplement: S3 File — (DOCX) [file pone.0238851.s003.docx]

Abstract presented at the RESNA Annual Conference 2018.

**Available at:** [**https://www.resna.org/sites/default/files/conference/2018/outcomes/Augustine.html**](https://www.resna.org/sites/default/files/conference/2018/outcomes/Augustine.html)

**DEVELOPMENT OF A MINIMUM UNIFORM DATASET (MUD)**

**FOR INTERNATIONAL WHEELCHAIR SERVICE AND PROVISION**

Nancy Augustine, Ms.Ed., *University of Pittsburgh*; Padmaja Kankipati, Ph.D., *Specialized Mobility Operations and Innovation Pvt. Ltd, India*; Jonathan Pearlman, Ph.D., *University of Pittsburgh*; Karen Rispin, *LeTourneau University*; Richard M. Schein, Ph.D., MPH, *University of Pittsburgh*

**ABSTRACT**

The International Society of Wheelchair Professionals (ISWP) developed long- and short-form versions of a Minimum Uniform Dataset (MUD) questionnaire which service providers can use during wheelchair service and provision around the world. Data can be gathered for a client who is being evaluated to receive a wheelchair for the first time or for a replacement wheelchair.  The questionnaire enables service providers to collect data as part of their standard of practice, such as those recommended by the World Health Organization (World Health Organization, 2012) and RESNA (Arlege et al., 2011). Service providers also will have the option to share de-identified data from the questionnaire with ISWP so aggregated information is available to support service providers and clients in improving wheelchair provision and service internationally.

**BACKGROUND**

The International Society of Wheelchair Professionals’ (ISWP) mission is to serve as a global resource for wheelchair service standards and provision through advocacy, education, standards, evidence-based practice, innovation and a platform for information exchange. The organization is currently based at the University of Pittsburgh and funded by the United States Agency for International Development (USAID). Among ISWP’s goals are to: a) provide service providers with tools to collect data as part of their standards of practice to help determine how they are contributing to improved wheelchair service provision; and b) encourage service providers to share de-identified minimum data so that, when aggregated, ISWP can use to benefit service providers and clients.

A Minimum Uniform Dataset (MUD) is common among most clinical professions to help build large-scale datasets, but a standard tool has yet to be developed for wheelchair service provision, which is the process through which an individual receives an appropriate wheelchair. Wheelchair service provision forms included in the World Health Organization (WHO) Wheelchair Service Training Package-Basic (WSTP-B) (World Health Organization, 2012) and University of Pittsburgh Department of Rehabilitation Science and Technology patient-reported outcomes (Kumar et al, 2011), for example, provide a solid foundation on data to be collected but may lack domains that are important to global partners. The RESNA “Wheelchair Service Provision Guide” describes the value of outcomes measures to “...document changes in the individual that would necessitate changes to the wheelchair and system” and cited several sample instruments (Arlege et al., 2011, p. 9). A 2012 review of wheeled mobility service delivery and outcomes measures, as well as discussions with subject matter experts, included recommended elements of service delivery but noted research gaps and a need for additional research across a variety of settings (Greer, Brasure, & Wilt, 2012).

This paper describes the objective of the MUD for international wheelchair service and provision, methodology, outcome and plans for future work.

**OBJECTIVE**

The objective of ISWP’s Minimum Uniform Dataset is to provide a survey instrument which service providers can use in their daily practice to gather information about wheelchair users’ needs and inform wheelchair service and delivery. The survey includes some fields that correspond to the WHO 8 Steps for basic wheelchair provision forms (World Health Organization, 2012), which many international service providers use, to avoid duplicating efforts during client visits. ISWP members are encouraged to use the questionnaires in their practices and share de-identified data with ISWP.

**METHODOLOGY**

ISWP used an iterative approach to gather feedback, then develop and pilot the tool with stakeholders from around the world.

ISWP Member Survey

On May 5, 2015, ISWP sent an e-mail to 353 ISWP members who are wheelchair sector stakeholders with a survey link to request input on: Data they currently collect in their practices; methods of collecting the information; willingness to share de-identified data to help develop common data fields and use a standardized data management system; methods for using de-identified, aggregated data; and suggested data to be collected. Forty-one individuals (41) responded, for an 11.6% response rate.

Among 39 respondents who reported their occupation, 28% (n=11) were clinicians; 15% (n=6) worked for non-government organizations; 15% (n=6) were researchers; and 10% (n=4) were manufacturers. Thirty percent (n=12) were grouped in the Other category, which was comprised of suppliers (n=3), academicians (n=3), as well as individuals who reported to be in private practice, product manager, or technician.

Slightly less than half of respondents -- 47.4% (n=18) -- indicated they collected data on wheelchair skills and abilities of their clients; 52.6% (n=20) did not. Among the methods used and reported by 36 respondents (multiple responses accepted): 77.7% (n=28) kept records on paper; 72.2% (n=26) conducted user interviews at time of delivery or follow-up (data collection method not specified); and 58.3% (n=21) conducted user satisfaction, feedback or impact surveys.

Questionnaire Development

In the 2015 survey, respondents identified domains they felt should be included in a minimum uniform data set. Eighteen data elements that were mentioned by 80% of respondents (n=33) were considered to be included. The ISWP Evidence-based Practice Working Group Data Collection Subcommittee, comprised of a cross-section of wheelchair sector stakeholders, used these data points as the basis for the first draft of the MUD. The subcommittee also reviewed the WHO wheelchair service provision forms in the WSTP-B (World Health Organization, 2012), data fields which World Vision was collecting as part of its ACCESS project (World Vision, 2015), WHO Quality of Life (World Health Organization, 1996) and Disability Assessment Scale (World Health Organization, 2017) measures, among other data points they felt service providers should know to inform wheelchair service and provision in international settings. As the number of data points to be collected grew, the Data Collection Subcommittee identified what minimum information should be collected so that a variety of organizations could share the same data points with ISWP.

In late 2015 and early 2016, the Subcommittee fine-tuned the questionnaire in preparation for a May 2016 pilot in Kenya. The purpose of the pilot was to determine: a) how long it took to administer the questionnaire; b) background of the individual administering it; c) setting/location where it was administered; and c) whether the wording was at a suitable level for comprehension.

2016 Pilot

A pilot was conducted in May 2016 in Kenya by Karen Rispin, associate professor of biology, LeTourneau University, with 45 primary school students (average age 11 1/2 years) and 60 secondary school students (average age 17 years), all wheelchair users. Data collection was done as part of a study for which ethics approval was given by LeTourneau University and LeTourneau’s partner organizations in Kenya. Consent and assent forms for all participants are on file at LeTourneau University. Participants completed paper versions of the questionnaires.

At the primary school, 45 wheelchair users came to a room individually to have their wheelchairs assessed by two physical therapists with extensive wheelchair experience. After the assessment, a single data collector administered the ISWP MUD to all 45 wheelchair users. An occupational therapist working with the students reviewed the completed documents to identify if there were inaccuracies, specifically with regard to diagnosis. At the secondary school, 60 wheelchair users completed the form in a group session. The research team circled the room to try to clarify questions while students completed the MUD.  One of those present was a teacher who was familiar with most students’ diagnoses and helped participants with that question. Those administering the questionnaire recorded comments on an Excel spreadsheet about difficulties with specific questions (e.g., very few respondents knew their height and weight or the year of diagnosis; most countries use meters to measure distance, not feet).

The 2016 pilot feedback and additional Working Group and Subcommittee input resulted in a standard 36-question version of the MUD and a 26-question short form. The standard version has additional questions about wheelchair and cushion manufacturer, make, and model; training received; assistance using a wheelchair indoors and outside; distance traveled in the wheelchair; whether the client takes public transportation; and which transportation methods are used. ISWP also created Excel workbooks for each version which included drop-down boxes for questions with close-ended responses to facilitate data entry.

2017 Pilots

A second pilot of the short form questionnaire was conducted by LeTourneau University in Kenya in May 2017 with 31 primary school students and 64 secondary school students, all wheelchair users. A volunteer used the Excel workbook to administer the questionnaire with the primary students. Secondary school students completed the paper questionnaire with help from volunteers who clarified questions, but did not help with responses, and noted when students said they had difficulty completing a question. Secondary school students took, on average, 11 minutes to complete the questionnaire and had difficulty answering 7 of the 26 questions.

In February 2017, UCP/Wheels for Humanity partnered with Massachusetts Institute of Technology (MIT) to pilot a standard version of the survey (36 questions) with 150 respondents in Bali, Indonesia as part of the Google User’s Voice Project. The questionnaire was translated and administered in Bahasa. MIT provided feedback to ISWP in December 2017. Overall, the MIT team felt the questions were useful but noted several items which were difficult for respondents to answer; e.g., it was not easy for users to remember when they received their chair, but they could give a date range; users could not easily remember when they received their diagnosis but could estimate the number of years; and some questions were double barreled. They also revised some questions to better meet their study objectives.

Finalizing the Questionnaires

Feedback from the 2017 pilots was incorporated, along with input from domain experts in instrument development at the University of Washington and University of Pittsburgh. The Evidence-based Practice Working Group provided additional feedback, resulting in final versions of the questionnaires prepared in March 2018 and presented to the ISWP Evidence-based Practice Working Group on April 5, 2018. The Excel workbooks and interviewer guides also were updated.

**OUTCOME**

Two versions of the minimum dataset questionnaire are available: A short form, with 26 questions, and a standard version, with 36 questions. The standard version has additional questions about wheelchair and cushion manufacturer, make, and model; training received; assistance using a wheelchair indoors and outside; distance traveled in the wheelchair; whether the client takes public or private transportation; and which transportation methods are used. Excel workbooks and interviewer guides for each version also are available to assist in data collection. Table 1 describes data included in each questionnaire.

An April 2017 version of the short form is available in French.  The translation was provided by Université de Montréal, CHU Sainte-Justine Centre de réadaptation Marie Enfant.  It has not been forward-backward-forward translated, and there have been several changes to questions which are reflected in the 2018 short form.

ISWP also explored a variety of open data kits which can accommodate the questionnaires; ultimately, organizations using the MUD are in the best position to determine if an open data kit meets their needs.

*Table 1: ISWP MUD Questionnaires (bold X indicates data point in standard version only)*

| **Data** | **Short Form** | **Standard Version** |
| --- | --- | --- |
| Client and wheelchair clinic information | X | X |
| Client ID |  |  |
| Client country |  |  |
| Wheelchair clinic ID |  |  |
| Wheelchair clinic country |  |  |
| Date completed | X | X |
| Purpose of Visit | X | X |
| Name of individual/organization referring client |  | **X** |
| Demographics | X | X |
| Age/approximate age |  |  |
| Gender |  |  |
| Education |  |  |
| Employment |  |  |
| Living situation |  |  |
| Reasons for Assistance |  |  |
| How long need something to help walk/move | X | X |
| Diagnosis | X | X |
| Year diagnosis received | X | X |
| Difficulty walking long distance (100 meters) | X | X |
| Assistance when outdoors/inside |  | **X** |
| Items used to help walk/move (list) |  |  |
| Use indoors/outside | X | X |
| Use more than one year | X | X |
| Number of days/week used | X | X |
| Number of hours/day used | X | X |
| Questions for current wheelchair users |  |  |
| Degree of difficulty pushing | X | X |
| How client typically pushes wheelchair | X | X |
| If not pushing with arms reasons why | X | X |
| Places where wheelchair is used | X | X |
| Whether public/private transportation is used |  | **X** |
| If public/private transportation used, what kind |  | **X** |
| Whether client has ever fallen out of wheelchair |  | **X** |
| How person received wheelchair | X | X |
| Agreement statements regarding wheelchair | X | X |
| Level of satisfaction with wheelchair | X | X |
| Wheelchair training received |  | **X** |
| Manufacturer name, model, serial number of item used to help person walk/move |  | **X** |
| Manufacturer name, make, model name of cushion and/or postural support device(s) used |  | **X** |

**FUTURE WORK**

Future work includes establishing the psychometrics of the MUD and performing formal construct and test-retest validity. Furthermore, validation of translated versions of the MUD are necessary for the French and upcoming Spanish versions of the tool.

There also are opportunities to develop the data collection and sharing system. The 2015 survey included a question which asked whether respondents would be interested in collecting shared data using a standardized data management system. Two-thirds (65.9%) of respondents (n=27) expressed interest; the remaining 34.1% (n=14) indicated “maybe.” Respondents also were asked to rate a list of electronic data collection system features on a Likert scale ranging from very important to very unimportant. Features which were identified as very important were: Easy to use interface (n=28); confidentiality of data (n=26); ability to easily create reports, charts and graphs from the data set about the organization’s impact (n=23); and technology designed for developing countries (n=21).

Respondents indicated they would use the aggregated data in a number of ways, including: Evaluating impact of service provider’s work (n=34); collaborating with other organizations (n=33); planning for future work (n=32); quantifying the service provider’s contribution to the sector (n=30); and, similarly, benchmarking (n=29).

The data collection and sharing system presents an opportunity to aggregate de-identified data for additional analyses across a variety of contexts. The system is included in ISWP’s strategic plan and will be developed as future funding permits.

**ACKNOWLEDGMENTS**

The authors thank the ISWP Evidence-based Practice Working Group, Data Collection, and Comparative Effectiveness Research Subcommittee members for helping to develop the questionnaires: Kavi Bhalla, Johns Hopkins University; Johan Borg, Lund University; Nathan Bray, Centre for Health Economics and Medicine Evaluation; Molly Broderson, Free Wheelchair Mission; Mark Harniss, University of Washington; Kristi Haycock, LDS Charities; Maria Toro Hernandez, Universidad CES; Astrid Jenkinson, Motivation UK; Sara Munera, WHEE; Karen Reyes, WHO; Chandra Whetstine, World Vision; and Eric Wunderlich, LDS Charities. We thank LeTourneau University staff, BethanyKids Relief and Rehabilitation, Joy Town School for Students with Disabilities, and the Google User’s Voice Project, led by UCP/Wheels for Humanity and implemented by MIT, for piloting the questionnaires. We also thank University of Montreal representatives Paula Rushton and Geneviève Daoust for the French translation of the short form and University of Pittsburgh faculty and staff Deepan Kamaraj, Krithika Kandavel, Patricia Karg, and Christina Zigler.

**REFERENCES**

Arlege, S., et. al. (2011). *RESNA Wheelchair Service Provision Guide*. Arlington, VA.: Rehabilitation Engineering & Assistive Technology Society of North America. (ED534426)

Greer, N., Brasure, M., & Wilt, TJ. (2012). Wheeled Mobility (Wheelchair) Service Delivery: Scope of the Evidence. *Annals of Internal Medicine*, 156, 141-146. doi: [10.7326/0003-4819-156-2-201201170-00010](https://doi.org/10.7326/0003-4819-156-2-201201170-00010)

Kumar, A., et. al. (2012) Test-retest reliability of the functional mobility assessment (FMA): a pilot study, *Disability and Rehabilitation: Assistive Technology*, 8:3, 213-219. doi: [10.3109/17483107.2012.688240](https://doi.org/10.3109/17483107.2012.688240)

World Health Organization (2017). *WHO Disability Assessment Schedule 2.0 (WHODAS 2.0).* Retrieved from: <http://www.who.int/classifications/icf/more_whodas/en/>

World Health Organization (2012). *Wheelchair Service Training Package-Basic Level*. Retrieved from: <http://www.who.int/disabilities/technology/wheelchairpackage/en/>

World Health Organization (1996). *WHOQOL-BREF*. Retrieved from: <http://www.who.int/mental_health/media/en/76.pdf>

World Vision ACCESS Project (2015). [Data fields]. Provided by World Vision.
