# Supplementary material for: Preliminary steps of the development of a Minimum Uniform Dataset applicable to the international wheelchair sector
Source: PLoS One. 2020 Sep 11;15(9):e0238851. doi: 10.1371/journal.pone.0238851 (PMC7485892; doi:10.1371/journal.pone.0238851)
Supplement: S2 Dataset — (DOCX) [file pone.0238851.s005.docx]

**S2 Dataset. Data collectors’ feedback after piloting the MUD in Kenya in 2016 and 2017**

**MUD Feedback**

**Kenya Pilot 2016**

#8 Other. Seemed Unnecessary- Pull Down Sufficient

#9. Most participants knew year of birth but not age- Interviewer Calculated

#12 Other. Seemed Unnecessary- Pull Down Sufficient

#13 Other. Seemed Unnecessary- Pull Down Sufficient

#14. Most participants did not know how long the mobility aid was needed for- Interviewer based answer on information given by a therapist

#15. Most participants did not know their diagnosis- Interviewer answered question based on information given by a therapist

#15 Other. Seemed Unnecessary- Pull Down Sufficient

#16. Most participants did not know their year of diagnosis- Interviewer based answer on their year of birth

#17a. Nobody knew the type of wheelchairs the participants were currently in

#17c. Some participants did not know how long they were in their current wheelchairs- Interviewer based answer on information given by a therapist

#18. Interviewer changed question and asked participants, “Can you walk – yes, kind of, or no?”

#19. Interviewer changed question and asked participants, “Is it easy, hard, or can you not push yourself?”

#20 Comment. Seemed Unnecessary-Pull Down Sufficient

#21 Other. Seemed Unnecessary- Pull Down Sufficient

#22 Transportation. Interviewer misunderstood question- Where the interviewer is from, people sit in their wheelchairs while riding public transportation. So the interviewer asked participants, “Do you sit in your wheelchair while riding public transportation?”-yes or no.

#22 Other. Seemed Unnecessary- Pull Down Sufficient

#23 Other. Seemed Unnecessary- Pull Down Sufficient

#24a. Interviewer changed question and asked the participant, “Does your wheelchair meet your needs- yes, no, or kind of?”

#24b. Interviewer changed question and asked participant, “Does your wheelchair work- yes, no, or kind of?”

#24c. Interviewer answered question based on looking at the participant in their current wheelchair

#24d. Interviewer changed question and asked participant, “Is it easy to push yourself- yes, no, or kind of?”

#24e. Interviewer required an English to Swahili translator. Also, interviewer changed question to have a yes, no, or kind of response.

#24f. Interviewer responded to question by personally feeling the cushion of the wheelchair that the participant was currently sitting in.

#24g. Interviewer changed question and asked the participant, “Does the cushion (of your current wheelchair) hurt to sit on (ie. do you get sores)- yes, no, kind of?”

#25. Interviewer asked participant, “Do you like your current wheelchair- yes, no, kind of?”

#25 Comment. Interviewer only added if participant provided information or feedback.

**Kenya Pilot 2017**

The comments mainly reflect removal of Other and Comments throughout, and simplifying some questions and reducing the number of responses from a number/likert scale to yes/no/somewhat.

Recommendations to be discussed with the Evidence Based Working Group:

1.       Remove Other and Comments from all questions

2.       Simplify question #18 to “In the past 30 days, have you had difficulty walking a long distance?” and reduce number of responses to three (yes, no, somewhat)

3.       Simplify question #19 “Is it difficult to push your current wheelchair?” and reduce number of responses to three (yes, no, somewhat)

4.       Change “Transportation” response in #22 to “Sit in wheelchair on a vehicle” or similar

5.       Reduce number of responses for 24a-f to four (yes, no, somewhat, don’t know)

6.       Reduce number of responses for #25 to yes, no, somewhat

- My main observation is that the interviewer/clinician interviewed a group of younger students.  He or she simplified the wording for the children interviewed in multiple places.  Instead of difficulty scales with 5 or 6 options “none // mild // moderate // severe // extreme // no answer”, he or she simplified it further to asking the patient “is it easy, hard, or can you not perform this task?”  (ex: 19) If possible, I think it could be beneficial to keep the rankings simply worded like this.
- Similarly, the interviewer modified the questions that require a “disagree - agree” 7-option scale to 3 choices: “Yes,” “no,” or “kind of” (ex: 18, 24a-24d). Again, if possible, this simplification could help clinicians complete the MUD set.
- The feedback says multiple times that the “other” option wasn’t necessary and that the options given sufficed but I don’t feel there is any reason to get rid of “other” anywhere even though the options are thorough.
- Number 22 the meaning of option “transportation” is unclear.

**Data collectors notes taken while administering the MUD**

| **Client Provided** | **Therapist Provided** | **Interviewer Provided** | **Not Completed** |
| --- | --- | --- | --- |
| Q1. Name | Q3. Client Town | Q2. Client ID | “Other “Responses |
| Q6. Service Provider Location | Q4. Client Country | Q7. Date Completed | Q17. Mobility Aids #2 on |
| Q9. Age | Q5. Service Provider Name |  |  |
| Q10. Gender | Q8. Visit Reason |  |  |
| Q11. Education | Q14. How Long Mobility Aid Needed |  |  |
| Q12. Employment | Q15. Reason for Mobility Aid |  |  |
| Q13. Living Situation | Q23. Current wheelchair source |  |  |
| Q16. Diagnosis Year |  |  |  |
| Q17. Mobility Aid #1 (Where used, used more than one year, hours per day used, days/week used) |  |  |  |
| Q18. Can you walk |  |  |  |
| Q19. Difficulty pushing wheelchair |  |  |  |
| Q20. How push wheelchair |  |  |  |
| Q21. Reason for not pushing wheelchair |  |  |  |
| Q22. Where wheelchair used (home, school, work, other public places, transportation) |  |  |  |
| Q24. Agreement statements |  |  |  |
| Q25. Satisfaction rating |  |  |  |
| Total: 16 | Total: 7 | Total: 2 |  |
