# Supplementary material for: Preliminary steps of the development of a Minimum Uniform Dataset applicable to the international wheelchair sector
Source: PLoS One. 2020 Sep 11;15(9):e0238851. doi: 10.1371/journal.pone.0238851 (PMC7485892; doi:10.1371/journal.pone.0238851)
Supplement: Attachment — Submitted filename: PLoS_one_04.07.20.docx [file pone.0238851.s006.docx]

# Development of a Minimum Uniform Dataset applicable to the international wheelchair sector

Rehabilitation outcomes are compared through time with large datasets, but use of assistive technology at discharge is not recorded outside the US, and outcome measures are not comparable between sites/countries. A uniform dataset is needed to help lower and middle income countries improve rehabilitation outcomes with evidence-based practice.

**1. Is the manuscript technically sound, and do the data support the conclusions?**

The manuscript must describe a technically sound piece of scientific research with data that supports the conclusions. Experiments must have been conducted rigorously, with appropriate controls, replication, and sample sizes. The conclusions must be drawn appropriately based on the data presented.

Yes

**2. Has the statistical analysis been performed appropriately and rigorously?**

I don’t know

**3. Have the authors made all data underlying the findings in their manuscript fully available?**

No

**4. Is the manuscript presented in an intelligible fashion and written in standard English?**

Yes

**5. Review Comments to the Author**

# General comments

The text contains a lot of jargon that people outside this area would be unfamiliar with – wheelchair sector, wheelchair stakeholders, etc. – that should be defined in the introduction. I am in the SCI field and I’ve never heard the term “wheelchair sector” and I’m unclear as to who a stakeholder is. Also, if “wheelchair sector” refers to individuals what use wheelchairs for mobility, it should be replaced with people-first language (“individuals that use wheelchairs” etc.).

The introduction could benefit from restructuring/reorganization: it’s long and there’s a lot of (necessary) information presented, but it’s hard to keep track of the aims of the paper and some of the ideas are repeated in each paragraph. For example, you talk about datasets in paragraphs 1 and 3, use of AP is introduced in paragraphs 1, 2, and 3, LMICs are discussed in paragraphs 2 and 3, and that there is no comparable research for clinicians to use is discussed in paragraphs 1 and 3. It would be easier to follow/understand if the structure were first paragraph – unmet need/significance; second paragraph: current datasets/evidence available; third paragraph: what is missing from datasets and why is it necessary.

Methods – there should be a sentence that summarizes this methodology in the beginning (needs assessment, rounds of pilot testing, refinement, etc.) with citations so the reader can know this methodology is standard for development and application of questionnaires/surveys.

Regarding the discussion – there was a lot of emphasis in the introduction on AP but no mention of it in the discussion. If it’s necessary and now included in the dataset, how is that going to improve the lives of individuals with SCI? How is that going to change rehabilitative outcomes?

# Specific comments

31 Rehabilitation is a strategy to promote functioning and a prerequisite to achieve health and well
32 being for all (1)

“functioning” is awkward, and the tense doesn’t agree with the rest of the sentence – maybe “functional gains” or something similar is better

41 outcome measures which are useful to compare rehabilitation outcomes among type of services

“which are useful” should be replaced with “in order to”

46 hospitalization, diagnostic, functional status data, but do not measure assistive technology use
47 at discharge (7, 8, 10, 12). The use of assistive products (AP) has been identified globally as an

The abstract and line 46 have “assistive technology” but the abbreviation is for assistive products – should be consistent throughout

52 For many people, access to AP, and learning how to use them, is needed to improve functioning

Change “functioning”

67 rehabilitation services both in quantity and quality (4). A full picture of the wheelchair sector is
68 necessary for strategic planning and the capacity building to realize the right to personal mobility

“a full picture” of what? AP use/needs?

69 (15, 30). In fact, a global wheelchair sector stakeholders meeting in 2018 identified as one of the
70 sector’s 10 priorities to:

Who is in the sector and who is a stakeholder? What was the meeting?

74 to support current recommendations on wheelchair service delivery models, and the evidence

Recommendations by whom? What is a wheelchair service delivery model?

79 measurements and had high attrition rates, making it impossible to aggregate the data (36-39).

I don’t think I’ve ever seen attrition used in this manner and I’m unclear – turnover is high among individuals administering the assessment? Or are individuals that participate lost to follow-up?

97 To help bridge the gap in wheelchair provision data gathered and comparable in LMIC, the
98 International Society of Wheelchair Professionals (50) developed a Minimum Uniform Dataset
99 with the objective to promote the global use of a common language to strengthen the evidence
100 (29) and to foster comparison among interventions (33).

Something similar to this statement should be moved earlier in the introduction, there’s a lot of background presented and it’s very easy to lose sight of the message

101 present the development process and final composition of ISWP’s

I’m assuming this is an abbreviation for International Society of Wheelchair Professionals but it wasn’t defined on line 98

117 On May 5, 2015, ISWP sent an e-mail to 353 ISWP wheelchair sector stakeholders who are ISWP […]
121 identified, aggregated data; and suggested data to be collected. Forty-one individuals (41)
122 responded, for an 11.6% response rate.

Is this typical survey method and a typical response rate for this type of analysis? It seems low and maybe would have benefitted from multiple emails/methodologies/etc. How can we be sure this is a representative sample of clinicians/professionals necessary to make the standards valid?

137 satisfaction, feedback or impact surveys

Need comma after “feedback”

197 The 2016 pilot feedback and additional working group and subcommittee review and input
198 resulted in a standard version of the MUD with 40 data points and a short form with 28 data point

It’s unclear why a short form and standard form were considered necessary

233 and some questions were double barreled.

What is “double barreled”?

238 Feedback from the 2017 pilots was incorporated, along with input from domain experts in
239 instrument development at the University of Washington and University of Pittsburgh.

A citation of some sort would be useful here, to demonstrate this is the standard method for modifying survey questionnaires based on pilot data

271 tool of its kind intended to promote global wheelchair service data collection and developed
272 iteratively with input from global stakeholders

Again – it’s unclear who the stakeholders are, and what “wheelchair service” refers to

274 has been identified as key for the success (8, 56).

Are you saying it’s already been successful? Or the collaboration will make it successful?

279 disaggregated by disability (5, 22). In addition, as the global wheelchair sector strives to set
280 service standards (31) […]
287 to illustrate the factors that affect the cost effectiveness of wheelchair provision (58).

These lines are all related to the same idea (standards/development of best practices), it can be decreased to one sentence. It’s speculation

294 feasible. In addition, preliminary data from the registry in the US has shown that administering

“shown” should be replaced with a more quantitative word

296 the clinical routine (44). The dataset being used in the US has 31 questions as compared to 29
297 questions in the short version MUD and 38 questions in the standard version MUD (44).

Why is the dataset different?

303 has accompanying written instructions on how to use it. Future work is needed to maximize data
304 quality.

What specifically needs to be improved and why?

312 A potential limitation that this MUD presents is that it is a self-report measure, which is expected

It seems a limitation is also the small response during creation of the questionnaire by stakeholders, and the fact that implementation is mostly limited to ISWP stakeholders. How do the authors propose to get this dataset used by a wider audience?

332 grass roots organizations of people with disabilities who can contribute to gathering, monitoring
333 and evaluating data points in addition to providing users’ perspective (22, 27).

Is improving their own care and health outcomes really an appropriate burden to give individuals with SCI? It is beneficial to understand their input and use it to guide best practices, but it’s not the responsibility of marginalized/unempowered individuals to correct the system that disenfranchises them.
